# The Application of Converter Sludge and Slag to Produce Ecological Cement Mortars

**DOI:** 10.3390/ma17174295

**Published:** 2024-08-30

**Authors:** Malgorzata Ulewicz, Jakub Jura, Adam Zieliński, Jacek Pietraszek

**Affiliations:** 1 Faculty of Civil Engineering, Czestochowa University of Technology, Dabrowskiego 69 Street, 42-201 Czestochowa, Poland; jakub.jura@pcz.pl; 2 Łukasiewicz Research Network—Upper Silesian Institute of Technology, 44-100 Gliwice, Poland; adam.zielinski@git.lukasiewicz.gov.pl; 3 Faculty of Mechanical Engineering, Cracow University of Technology, Al. Jana Pawla II 37, 31-864 Kraków, Poland; jacek.pietraszek@pk.edu.pl

**Keywords:** metallurgical waste, sludge, slag, cement mortar, mechanical engineering

## Abstract

The paper presents an analysis of the effective use of a mixture of steel sludge (S1) and slag (S2) from the converter process of steel production for the production of cement mortars. Metallurgical waste used in the research, which is currently deposited in waste landfills and heaps near plants, posing a threat to groundwater (possibility of leaching metal ions present in the waste), was used as a substitute for natural sand in the range of 0–20% by weight of cement (each). The obtained test results and their numerical analysis made it possible to determine the conditions for replacing part of the sand in cement mortars with a mixture of sludge and slag from a basic oxygen furnace (BOF) and to determine the effects of such modification. For the numerical analysis, a full quadratic Response Surface Model (RSM) was utilized for two controlled factors. This model was subsequently optimized through backward stepwise regression, ensuring the inclusion of only statistically significant components and verifying the consistency of residual distribution with the normal distribution (tested via Ryan-Joiner’s test, *p* > 0.1). The designated material models are helpful in designing ecological cement mortars using difficult-to-recycle waste (i.e., sludge and converter slag), which is important for a circular economy. Mortars modified with a mixture of metallurgical waste (up to 20% each) are characterized by a slightly lower consistency, compressive and flexural strength, and water absorption. However, they show a lower decrease in mechanical strength after the freezing–thawing process (frost resistance) compared to control mortars. Mortars modified with metallurgical waste do not have a negative impact on the environment in terms of leaching heavy metal ions. The use of a mixture of sludge and steel slag in the amount of 40% (slag/sludge in a 20/20 ratio) allows you to save 200 kg of sand when producing 1 m^3^ of cement mortar (cost reduction by approx. EUR 5.1/Mg) and will also reduce the costs of the environmental fee for depositing waste.

## 1. Introduction

Sustainable development of the construction sector is inextricably linked to the need to minimize the consumption of natural resources and optimal use of industrial waste for the production of various building materials. The issue of protecting sand and natural aggregate commonly used in construction and other industries is particularly important. The world industrial mining production of these materials in 2023, estimated at 400 million metric tons, increased by 10.3% compared to 2022 (359 million metric tons) [1]. The construction sector consumes the most sand, using approximately 50 million metric tons annually to produce mortars and concrete [2]. That is why it is so important to look for alternative materials that could replace, at least partially, natural raw materials in order to provide enough of them for future generations. In laboratory tests, a number of different wastes have been used as a substitute for natural aggregate in mortars and concretes, including glass cullet waste [3,4], thermoplastic elastomers [5,6], modified polymeric materials [7], ash from biomass combustion [8,9], or ash from sewage sludge combustion [10,11,12]. In addition, various types of urban waste, including plastic waste, used engine oil, and used glass, have also been used to produce sustainable concrete [13]. There have also been reports on the use of waste slags from non-ferrous metallurgy for this purpose, i.e., copper [14,15], nickel [16], zinc [17], and lead [18], and blast furnace slag from the steel industry [19,20,21] or a mixture of blast furnace slag and other waste [22,23]. The granulated blast furnace slag was also used as a substitute for natural silica sand in alkaline-activated slag mortar [24]. Unfortunately, other by-products (waste) generated in steelmaking processes, which differ significantly in their physicochemical properties, are not used in practice, and when moving towards a circular economy, it is worth considering these materials as an alternative replacement for natural aggregate.

The amount of by-products (waste) generated in the steel industry systematically increases as steel production increases. In 2021, the global production of crude steel amounted to 1951 million tons, of which 70.1% was produced in BOF (basic oxygen furnace) processes and 28.9% in EAF (electric arc furnace) processes [25]. It is estimated that, during the production process of 1 Mg of liquid steel in steel plants, an average of 0.6 Mg of various solid wastes is generated [26]. In the process of refining 1 Mg of pig iron using a basic oxygen furnace (BOF), 90–110 kg of oxygen furnace slag is produced [27,28] and approximately 20 kg of dust and sludge [29] and, from an electric arc furnace (EAF), 120–170 kg of slag and 15–20 kg of dust [30]. According to data from the World Steel Association [30], in Europe alone, 15.7 million tons of steel (converter) slag are produced, of which only about 40% are used, and the remaining part has no practical use and is deposited in landfills. Currently, steel slag in Europe is mainly used for road construction (approx. 69.9%) and in the metallurgical industry (13.4%). It is used, to a small extent, in the production of fertilizers (4.6%), cement/concrete (5.5%), hydraulic engineering (1.3%), and in other industries (total 5.3%) [31,32]. Therefore, the search for various other forms of management for approximately 60% of today’s steel slag is an important element in the economy heading toward a circular economy. It is also worth paying attention to other metallurgical waste, i.e., dust and sludge generated in the metallurgical process during steel production, which contains significant amounts of hazardous metals (i.e., As, Cd, Hg, and Ti) and is currently deposited in waste landfills and heaps at plants. Metals present in the waste, as a result of leaching, pose a risk of being released into the ground and groundwater, which poses a threat to the natural environment [33,34]. Taking into account the need to protect the natural environment, reducing the amount of space occupied by metallurgical waste deposited in landfills, as well as the need to introduce a circular economy, it seems advisable to conduct research on the use of waste materials from the steelmaking process as a replacement for sand (aggregate) in cement matrix composites.

The literature review shows that, for the production of cement-based composite materials in laboratory tests, the most frequently used waste materials from the steel industry were blast furnace slag, blast furnace ferronickel slag, and slag from electric arc furnaces. Other waste materials from this economic sector were not used or were used sporadically. Blast furnace slag [24,35] and granulated blast furnace slag [36,37] were used in cement mortars, both as an aggregate replacement and as a cement substitute [21]. The blast furnace slag was used as a cement substitute in mortars, also in combination with such materials as fly ash [38,39], hemp fiber [40], and silicone oil [41]. However, in concretes, granulated blast furnace slag was used as a cement substitute [42,43]. Granulated blast furnace slag and steel slag were also used as a substitute for aggregate in concrete [44,45], as an addition to concrete asphalt [46,47], and also as a replacement for cement in concrete [48,49]. The granulated blast furnace slag alone [50,51] or in combination with phosphogypsum waste was also used to produce cementless mortars [52]. There are also several reports in the literature on the use of granulated blast furnace slag in cement mortars in combination with other waste materials, such as red brick waste [21], silica fume [23], glass cullet [39], and fly ash [39,53].

In addition, blast furnace ferronickel slag [54,55], a mix of blast furnace ferronickel slag and blast furnace powder [56], electric arc furnace slag [57,58], steelmaking slag [59], and sludge [60] were also used in cement mortar. The electric arc furnace slag [61,62], metallurgical sludge [63,64], and steel sludge [65,66] were also used in concrete. However, according to the analysis of these publications, it is not possible to clearly determine the impact of the use of metallurgical waste on the properties of cement mortars and concretes, because it varies and is determined by the physicochemical properties of the added waste materials [67,68].

Noteworthy is the review work [31], where the author made a detailed analysis of articles posted on Web of Science (Core Collection databases) from 1990 to 2022 on the impact of alternative aggregate from steelmaking slag (from EAF and BOF processes and defined as steelmaking slag (SS), because the authors of the publication do not provide its source) on the properties of concrete and mortars. The analysis clearly shows that the authors demonstrate both positive and negative effects of slag aggregate on the properties of composites, such as workability, density, mechanical strength, water absorption, porosity, and chloride permeability, as well as resistance to sulfates, the freezing/thawing process, and carbonation. Analyzing the most frequently studied parameter, compressive strength, Rashad [31] reported that, in approximately 42.86% of the works, the authors showed a higher compressive strength of concrete with the addition of fine aggregate made of SS slag and, in 21.43%, lower compressive strength, and 35.71% of the works showed that adding fine slag aggregate to concrete may cause an increase or decrease in this parameter (depending on various factors, e.g., the degree of slag fragmentation and the amount of added waste). However, in the case of replacing coarse natural aggregate with steel slag, 76.47% of the authors reported an increase in the compressive strength of concrete, 17.6% a decrease, and 5.88% observed both an increase and a decrease in this parameter. There are also more critical opinions in the literature, showing that the use of metallurgical slags has a very unfavorable effect on the mechanical parameters of cement composites [17]. Therefore, it is not surprising that many researchers are still trying to answer the question of what waste from the steel industry and to what extent can be used for cement mortars and concretes without deterioration of their physical and mechanical properties. It should be emphasized that the physical and mechanical properties of composites produced using waste are determined by many factors, including the granulometric composition of the waste, the type of pre-treatment, its chemical composition, the amount of waste material introduced, the addition of plasticizers, and the presence of other waste.

This study analyzes the possibility of using two wastes from the steel industry to produce ecological cement mortars: sludge (S1) and slag (S2) from a basic oxygen furnace. According to the waste catalog (EU Decision 2000/532/EC), slag from the steel smelting process is classified in group 10 · 02 · 01 and sludge from waste gas purification containing hazardous substances in group 10 · 02 · 13* (asterisk indicates hazardous waste). The effective use of this waste to produce cement mortars fits into the idea of a circular economy. It is particularly important to use metallurgical sludge alongside slag due to the presence of heavy metal ions, which can be washed into the ground, posing a danger to humans and the environment when deposited in on-site heaps. The use of this waste to produce cement-based composites means that the metal ions present in the waste can be permanently incorporated into the structure of the cement matrix, minimizing the possibility of their leaching into the environment during the use of building materials.

## 2. Materials and Methods

### 2.1. Materials

**Figure 1:**
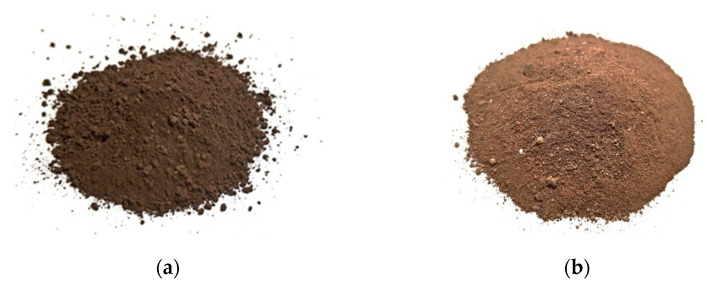
Waste sludge (**a**) and slag (**b**) from the oxygen furnace.

The tested mortars were produced using Portland cement CEM I 42.5 R (Cemex, Poland), meeting the requirements of the PN-EN 197-1 standard [69], “Kwarcmix” standard sand (with a fraction of 0–2 mm in accordance with the PN-EN 196-1 standard [70]), water from the intake in Czestochowa (meeting the requirements of the PN-EN 1008:2004 standard [71]), and waste from the metallurgical industry. Two metallurgical wastes used in the tests on the modification of cement mortars, sludge (S1) and slag (S2) from a basic oxygen furnace (Figure 1), coming from one of the Polish steel producers, are a by-product of the production process and are not used by the plant. Waste metallurgical sludge was dried in a dryer (temp. 105 °C for 24 h (dryer POL-EKO type SLW 240-W STD, Wodzisław Śląski, Poland) and then sieved. Materials with a grain size of <2 mm were used in the tests. The chemical composition of the metallurgical waste was obtained using an XRF X-ray spectrometer (Thermo Fisher Scientific, Waltham, MA, USA) and is presented in Table 1.

### 2.2. Research Methods

**Figure 2:**
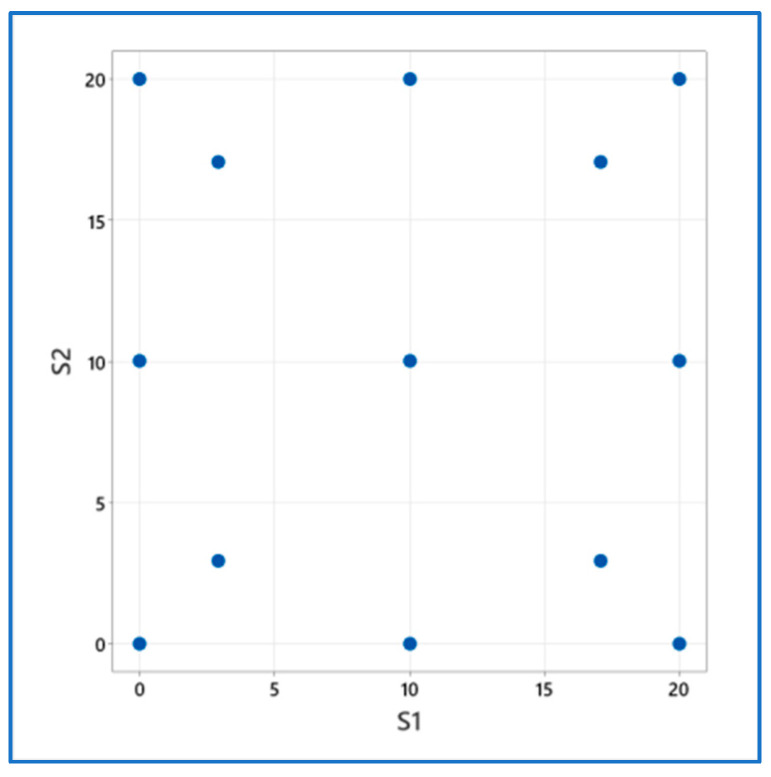
Arrangement of plan systems in the space of the experiment plan; S1 and S2 in [%].

The cement mortars used in the tests (control series and mortars modified with waste) were made in accordance with the EN 998 2:2016-12 standard [72]. The control mortar contained 450 g cement, 1350 g sand, and 225 g water. Waste metallurgical sludge (S1) and slag (S2) were used as a substitute for sand in the range from 0 to 20% by the weight of cement, according to the experimental design (Figure 2), which allows obtaining the maximum amount of information about the characteristics of mortars in the full analyzed range of variability of the controlled factors. Table 2 presents the assumed actual values of the controlled factors for the coded values and the experimental plan without replication.

The typical problem of modeling the observed quantity when postulating its dependence on controlled factors in the static variant, without considering the time factor and the dynamics of the process, can be realized in two ways. The first, taken into account when the resources at disposal allow for the implementation of only a relatively small number of tests and widely used by practitioners [73], consists of a specific arrangement of test points in the space of controlled factors by statistical algorithms postulated by the design of experiments (DOEs) classical methodology [74,75] and a priori imposition of the structure of the mathematical model, usually in the form of a polynomial of, at most second degree. Additional conditions resulting from conservation rules or general physical and chemical equations of the process can be taken into account, which is realized, among others, in the adjustment calculus [76,77]. The second case, available when the number of tests can be large, consists of the use of a data-driven approach, which can take the form of either a bottom-up creation of an analytical parametric model or—using machine learning methods—a nonparametric model. In this paper, the DOE approach was used due to limited resources and the high labor and time consumption of the tests. The experimental design was adopted as a composite design supplemented with corner points. Model identification and uncertainty estimation were performed using STATISTICA (TIBCO Statistica, version 13, Santa Clara, CA, USA).

Waste sludge (S1) and slag (S2) are the two controlled media. The observed values are consistency (C), flexural strength after 28 days (FS28), compressive strength after 28 days (CS28), water absorption (WA), weight loss after the frost resistance tests (ML), and decrease in flexural strength (DFS) and compressive strength (DCS) after the frost resistance tests. The initial experimental design was composed of a Box–Wilson central composite design (CCD) enhanced by adding four far corner points at the axial point levels. Ultimately, the controlled factors had 5 levels. The experimental design consisted of 13 different points, with 7 replications in the center, to determine the amount of random noise (pure error) from uncontrolled or weakly controlled factors. Using statistical methods to design the composition of concrete mortars significantly reduces research costs, time, and labor consumption while increasing the results’ reliability. The effectiveness of this approach has already been noticed in the design of steel-concrete structures [77] and should now also be promoted in mortar research.

The testing of the properties of cement mortars with dimensions of 4 × 4 × 16 cm was carried out in accordance with the relevant standards: PN-EN 1015-3 [78] (consistency), PN EN 1015-11:2020-04 [79] (compressive and flexural strength; MMC-3742 testing machine. FPR; ToniTechnik 2030, Berlin, Germany), and PN-85/B-04500 [80] (water absorption and frost resistance after 25 cycles of freezing and thawing using the Toropol K-010 climatic chamber; Toropol, Warsaw, Poland). In addition, the research also included an analysis of the leaching of metal ions based on the PN-EN-12457-2:2006 standard [81]. The analysis of the concentrations of leached metal ions was performed using an Agilent MP-AES 4200 microwave-induced plasma atomic emission spectrometer (Warsaw, Poland). SEM/EDS analysis of the surface microstructure of the synthesized mortars was also performed (LEO Electron Microscopy Ltd., Cambridge, UK, model 1430 VP).

## 3. Results

By-products from metallurgical processes of iron and steel production are increasingly becoming the subject of research interest in terms of the possibility of their utilization in building materials. For several years, blast furnace slag has been used in the construction sector [20,37]; produced in the production of pig iron (aggregate and cement additive); and in recent years, scientists have also focused on converter (steel) slag produced during steelmaking. This slag has been studied mainly in laboratories for use in concretes [32,65] and occasionally in mortars [57,58]. Steelmaking slags are recycled and used in both cement composites and other building materials [82,83]. Individual works also concern the use of other waste from the steelmaking process, i.e., sludge, in cement-based composites, i.e., cement mortar [60,84] and concrete [63,64].

In this research, converter slag was used together with steel sludge, classified as hazardous waste, in search of an effective method of its management. In the first stage of the research, the consistency, mechanical strength of hardened cement mortars, water absorption, mass loss, and mechanical strength were determined after the frost resistance tests. Then, 19 measured measurement results (Table 3) for each observed quantity were submitted for numerical analysis. In the second stage of the research, microscopic observations of the produced mortars and a leaching test of metal ions from the cement mortar matrix were carried out.

### 3.1. Consistency Analysis

The consistency (C) of the wet-mixed mortar that binds the bricks or aggregates together into a stable structure is crucial to ensuring the quality and strength of the product. Consistency affects workability (ease of spreading and shaping the mortar), adhesion (to bricks and aggregate), and strength (too little or too much water disturbs the proper setting process) [85]. A full quadratic RSM model was adopted to analyze the consistency of modified mortars for two factors (S1 S2 S1 × S1 S2 × S2 S1 × S2). The analysis of variance (ANOVA) showed that only the linear term for the S1 factor was statistically significant for the full quadratic model (Table S1). After reducing the model using the backward stepwise regression method, a model (S1 S1 × S2) was finally obtained in which both components are statistically significant. The model’s determination index was R^2^ = 0.73. The coefficients of the fitted model include the constant term (average response of the process), the linear term S1, and the two-way interaction term of a synergistic nature S1 × S2. The numerical formula is Form (1).

C = 14.99 − 0.715 · S1 + 0.362 · S1 · S2(1)

**Figure 3:**
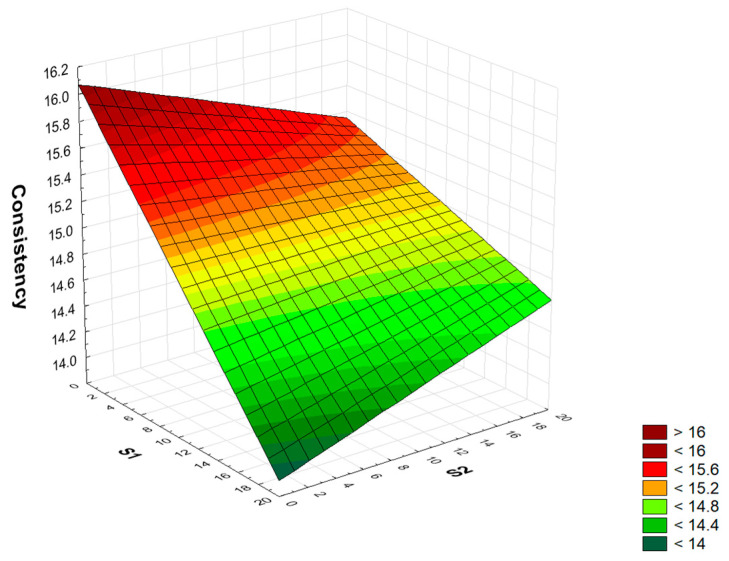
Graph of the dependence of the consistency [cm] of cement mortar on the amount of added sludge S1 [%] and slag S2 [%].

In its current reduced form, the model has only statistically significant components and model residuals consistent with the normal distribution (Ryan-Joiner test, *p* > 0.1), with a statistically significant lack of fit. A graph of the relationship between the consistency of cement mortars and the amount of added waste is shown in Figure 3. As demonstrated by the analysis, as the addition of sludge (S1) increases, the consistency of the produced cement mortars decreases, and synergistic interaction between S1 and S2 waste is observed. The addition of S2 slag in the range of 0–20% of the cement mass has no statistically significant effect on the consistency of the produced cement mortars. All cement mortars modified with the addition of waste sludge and slag have a lower consistency than the control mortar. The consistency measured using a flow table (PN-EN 1015-3) of the control mortar series was 15.9 cm, of mortars containing 20% sludge, 13.7 cm, and of mortars with 20% slag, 14.9 cm. Mortars containing the addition of a mixture of sludge and slag (20% each) have a flow rate of 14.3 cm. Therefore, the observed synergistic effect of the addition of a mixture of sludge and slag is beneficial and does not cause a rapid decrease in consistency (decrease by 10.1%), which is observed after the addition of the sludge itself (decrease by 13.8%).

A decrease in the consistency of mortars containing metallurgical waste (used as a replacement for natural aggregate) was also demonstrated [84]. Mortars containing 5, 10, 15, and 20% of sludge showed a decrease in consistency by 0.6, 2.5, 5.0, and 6.3%, respectively. However, mortars containing 5, 10, 15, and 20% of slag showed a decrease in consistency by 5.7, 7.5, 11.3, and 13.8% [84]. A decrease in the consistency of cement mortars was also observed after replacing natural aggregate with electric arc furnace slag (EAF) [57,58]. Santamaría et al. [57] showed that replacing natural aggregate at 60% with EAF (size < 5 mm) reduces the slump of the mortar mixture cone by 2.56%. At the same time, Santarmaría-Vicario et al. [58] reported a decrease in the workability of cement mortar mixtures after replacing natural fine aggregate with the amount of EAF released (grain size 0.063–2 mm). Replacement of sand in the amount of 25, 50, 75, and 100% by EAF reduces the cone slump by 21.61, 28.71, 40.32, and 72.26%, respectively. There was also an increase in the density of these mortars, with the increase in slag by 3.64, 6.88, 11.72, and 14.86%, respectively. However, Pizoń et al. [64] showed that the addition of 30 and 60% of steel sludge to concrete increases the consistency of concrete (the cone slump decreases from 105 mm to 20 and 10 mm, respectively). The opposite trend for concrete was observed by Qasrawi et al. [86], showing that replacing 15, 30, 50, and 100% of the aggregate with slag with a grain size of 0.075–5.0 mm reduces the cone slump by 8.33, 16.67, 20.0, and 83.33%, respectively. Despite numerous studies on the use of steel slag in concrete, as shown by Rashad [31], there is no clear answer regarding its effect on consistency. After replacing the natural aggregate with steelmaking slag (SS) or EAF slag, 69.7% of the authors of the works reported a decrease, 20.91% an increase in the consistency of concretes, and only in 9.3% of the works, no effect of this addition on the consistency of concretes was observed. These contradictory reports that metallurgical slag can increase or decrease the workability of concrete can be attributed to differences in the properties of the waste used by the authors to replace natural aggregate (the authors often do not provide the exact composition or source of origin of the waste used), different granulometric size of the waste, and different w/c ratio, as well as the degree of descent. It is worth emphasizing that steelmaking slag and sludge were also used in concrete as a substitute for cement [65]. Concretes in which the cement was replaced with 5, 10, 15, and 20% of slag obtained a consistency lower by 25, 37.5, 62.5, and 75%, respectively. However, replacing 5% of cement in concrete with sludge did not affect its consistency, while the addition of 10, 15, and 20% resulted in a reduction in consistency by 12.5, 37.5, and 75%, respectively.

### 3.2. Analysis of Standard Flexural and Compressive Strength

The work defines the material model of mortars modified with waste, understood as the quantitative relationship “mortar composition–mechanical properties”. To analyze the standard flexural (FS28) and compressive strength (CS28) after 28 days, the full quadratic RSM model for two factors (S1 S2 S1 × S1 S2 × S2 S1 × S2) was used. In the case of flexural strength, the analysis of variance showed that statistical significance was met only for the linear and quadratic terms of the S2 factor (see Table S2). After reducing the model using the backward stepwise regression method, a linear model (S2) was finally obtained, in which all terms were statistically significant. The model’s determination index was R^2^ = 0.60. The coefficients of the fitted model include a constant term (process mean response) and the linear term S2. The numerical formula is Form (2).

FS28 = 7.49 − 0.510 · S2 (2)

In its current reduced form, the model has only statistically significant components, the distribution of residuals is consistent with the normal distribution (Ryan-Joiner test; *p* > 0.1), and its accuracy is satisfactory due to the statistical insignificance of the lack of fit.

However, in the case of compressive strength, the analysis of variance showed that statistical significance was met for the full quadratic model only for the linear components of factors S1 and S2 (see Table S3). After reducing the model using the backward stepwise regression method, a linear model with a two-factor interaction (S1 S2 S1 × S2) was obtained. The model’s determination index was R^2^ = 0.80. The coefficients of the fitted model include a constant term (mean process response), two linear terms, S1 and S2, and a two-way interaction, S1 × S2. The numerical formula is Form (3).

CS28 = 45.80 − 2.493 · S1 − 5.45 · S2 − 3.61 · S1 · S2(3)

**Figure 4:**
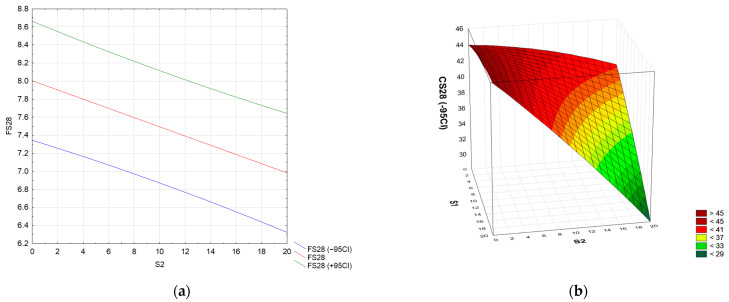
Graph of the dependence of the flexural strength [MPa] (**a**) and compressive strength [MPa] (**b**) of mortars after 28 days of maturation on the amount of added sludge S1 [%] and slag S2 [%].

In its current reduced form, the model has only statistically significant terms, and the distribution of residuals is consistent with the normal distribution (Ryan-Joiner test; *p* > 0.1). Unfortunately, its accuracy is unsatisfactory due to the statistical significance of the lack of fit. For the sake of prognostic caution, the graphs show the lower limit of the 95% predicted confidence interval (−95CI). Figure 4 shows a graph of the relationship between the flexural and compressive strength of the tested cement mortars after 28 days of maturation and the amount of metallurgical waste added. The flexural strength of cement mortars modified with the addition of a mixture of sludge and slag from the steelmaking process decreases linearly with the amount of added slag (Figure 4a). The flexural strength of mortars containing a mixture of sludge and slag (20% of each waste) is 18% lower than that of the control mortars. However, the resistance to dripping of mortars modified with this waste mixture decreases with the addition of sludge and slag, and an antagonistic interaction is observed for these wastes (Figure 4b). Waste-modified cement mortars exhibit compressive and bending strength required for M20 mortars, which can be used in the construction of foundations and load-bearing walls.

Mortars containing 20% slag show a decrease in compressive strength of 10.6% compared to the control samples, while mortars containing 20% slag show a 1.2% increase in their strength. The compressive strength of mortars containing a mixture of sludge and slag (20% of each waste) is 41.9% lower than that of the control mortars. The tendency we observed to reduce the mechanical strength of cement mortars after replacing the natural aggregate with a mixture of BOF sludge and slag is comparable to the tendency observed for mortars with the addition of EAF slag [61]. The authors showed that the compressive and flexural strength of mortars in which the aggregate was replaced with 50 and 100% EAF slag decreased after both 7 and 28 days of sample maturation. After 28 days, the flexural strength of mortars with the addition of 25 and 50% waste decreases by approximately 5 and 28%, respectively, and the compressive strength by 25 and 56%, respectively. In turn, Santamaría-Vicario et al. [58] reported a decrease in the early (after 7 days) compressive strength of mortars after adding EAF slag (grain size 0.063–2 mm) in amounts of 25, 50, and 75% by 0.79, 6.35, and 5.5%, respectively.

In the case of 100% replacement of fine aggregate with EAF slag, the authors observed an increase in this parameter by 5.55% compared to the control samples, while, after 28 days of maturation, mortars containing 25, 50, 75, and 100% of EAF slag showed higher compressive strength by 16.45, 17.22, 17.22, and 17.88%, as well as flexural strength by 23.68, 31.57, 31.7, and 34.21%. Öműr et al. [87] showed that the compressive strength of cement mortars containing BOF is lower than that of the control mortars. After 28 days, the compressive strength of mortars containing 50 and 100% BOF directly from production ranged from 46.4 to 40.0 MPa, while the strength of mortars containing weathered slag ranged from 39.1 to 33.1 MPa. Replacing the sand with 50 and 100% BOF (directly from production) and weathered sand reduced the compressive strength of the mortars by 3.0 and 19.8%. However, the flexural strength of mortars decreases slightly after 28 days after replacing the natural aggregate with BOF slag. Mortars containing 50 and 100 BOF directly from production were 6 and 7% lower, respectively, and mortars with weathered BOF slag were 7% and 15% lower than the reference mortar. The authors explain this by a better bond between the cement matrix and natural aggregate than slag. However, Santamaría et al. [57] showed that, after 28 days, the strength of the mortars in which the natural limestone aggregate was replaced by 60% with EAF slag, grain size < 5 mm, is 20% higher than the control mortars. Also, Ozturk et al. [88] showed a higher compressive strength of mortars in which fine lime-dolomite aggregate was replaced with EAF slag. After 28 days, for mortars with the addition of 20, 40, 60, 80, and 100% EAF, an increase in compressive strength of the mortars was recorded by 7.31, 13.24, 6.64, 4.82, and 7.8%, respectively. The research by Faraone et al. [59] reported that, with an appropriate w/c ratio, steelmaking slag (SS) of various grain sizes can replace fine aggregate in mortars without losing its compressive strength. Da Silva Magalhães et al. [89] showed that, with an increase in w/c from 0.35 to 0.7, the compressive strength of mortars containing both 5 and 10% EAF decreased from 33% to 34% after 28 days of sample maturation. Flexural strength also decreases by approximately 4%. However, in the case of using steelmaking slag (SS) or EAF as a substitute for concrete aggregate, as shown in the review [31], 76.47% of the publications report a decrease, 17.65% an increase, and 5.88% both a decrease and increase in compressive strength of concretes modified with this waste.

Steel sludge was added only to concrete [62,63,64,65], but in this case, both positive and negative effects of its addition were observed on the mechanical strength of manufactured materials. Roslan et al. [65] observed a greater decrease in compressive strength of concretes containing added sludge than of steelmaking slag used as a cement substitute after 28 days of curing. Only the standard strength for concrete containing 10% slag was higher (by approx. 7%) than for the control concrete. However, after 28 days, concretes containing 5, 15, and 20% of slag showed a decrease in standard strength compared to the control concrete. Also, concretes containing 5, 10, 15, and 20% of sludge reached lower compressive strength than the control mortars. The lowest decrease in strength was recorded for concrete containing 20% sludge (by approx. 40%). A similar trend was observed in the case of changes in the flexural strength. Concretes containing 10% slag showed a higher flexural strength (by approx. 5%), while concretes containing 20% sludge showed the lowest decrease in flexural strength by approx. 50%. Also, Alwaeli et al. [60] observed a decrease in the compressive strength of concretes in which natural aggregate was replaced with waste sludge in the amount of 20 and 30%. Only the compressive strength of concretes with the addition of 10% sludge was similar to the control samples. However, Pizoń et al. [64] observed an increase in the compressive strength of concrete with the level of replacing the aggregate with metallurgical sludge with a grain size of 0–0.25 mm. The compressive strength of concretes with the addition of 30, 60, and 90% of metallurgical sludge increased by 26.8, 29.1, and 2.2%, respectively, in relation to the control concrete. In turn, Lehner et al. [63] showed that the addition of 30% metallurgical sludge (grain size 0–0.25 mm) does not significantly improve or deteriorate the compressive strength of concrete after 28, 56, and 91 days of maturation (difference of approximately 2%). The use of the addition of 30% sludge in combination with an air-entraining admixture may slightly increase the compressive strength of concretes in relation to control concretes containing the admixture, which is probably caused by a different reaction of the air-entraining mixture to the replacement of fine aggregate. After 28 days, concretes with the addition of sludge and an air-entraining mixture showed higher strength by approximately 10% compared to the control concrete with an air-entraining mixture.

### 3.3. Water Absorption Analysis (WA)

A full quadratic RSM model for two factors (S1 S2 S1 × S1 S2 × S2 S1 × S2) was adopted for analysis. For the full quadratic model, the analysis of variance showed that statistical significance was met only for the linear term of the S2 factor (Table S4). After reducing the model using the backward stepwise regression method, a linear model (S2) was finally obtained, in which all terms were statistically significant. The model’s determination index was R^2^ = 0.75. The numerical formula for the coded factors is Form (4). The coefficients of the fitted model include a constant term (process mean response) and the linear term S2.

WA = 9.60 + 0.6752 · S2(4)

However, in the case of uncoded S2 values (range 0…20), the forecast formula is Form (5).

N = 8.92 + 0.06752 · S2(5)

**Figure 5:**
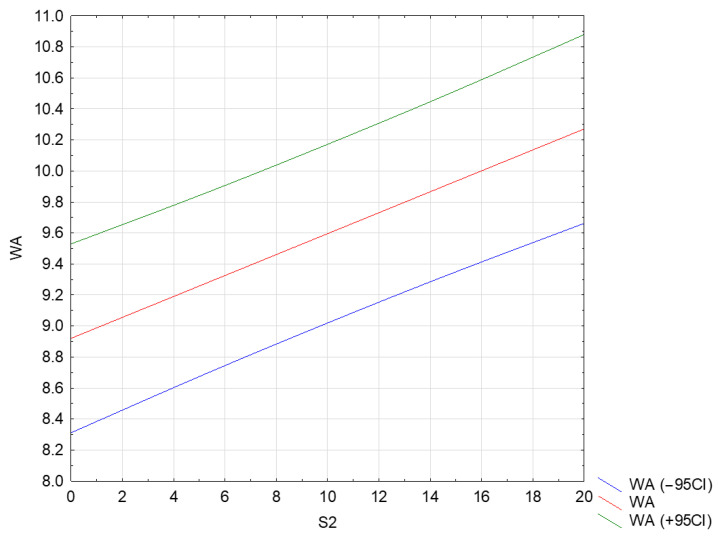
Dependence of water absorption [%] on the amount of slag S2 [%] in the mortar.

In its current reduced form, the model has only statistically significant terms, the distribution of the residuals is consistent with the normal distribution (Ryan-Joiner test; *p* > 0.1), and its accuracy is highly satisfactory due to the lack of statistical significance of the lack of fit (*p* = 0.991). Therefore, the predictive usefulness of this model is high. The plot predicts a linear relationship, with the water absorption increasing as S2 increases (Figure 5).

A scatterplot of the raw data gives an idea of the scatter of the experimental data. As can be seen from the graph, the water absorption of mortars modified with the addition of a mixture of BOF sludge and steelmaking slag increases linearly only with the addition of slag. Also, Öműr et al. [87] observed an increase in the water absorption of mortars with the addition of 50 and 100% of BOF slag coming both from direct production and from the heap (weathered/oxidized slag). The water absorption and pore volume of mortars containing BOF directly from production were lower compared to the mortar containing weathered waste for equivalent replacement factors. This is most likely due to the water absorption of these materials. BOF slag comes directly from production and is oxidized, which has higher water absorption by 4.46% and 11.33%, respectively, compared to natural sand. The water absorption of cement mortars with the addition of BOF ranged from 9.3 to 14.0%. For mortars made of 100% post-production and weathered slag, it was 11.5% and 14.0%. These results are largely consistent with those published by Bodor et al. [90], where the addition of BOF aggregate resulted in greater water absorption and porosity of the mortars. However, Santamaría et al. [57] reported that replacing fine aggregate in mortars at 60% with EAF slag (grain size < 5 mm) increased their porosity (1.3%) and water absorption. However, in the case of using steelmaking slag (SS) or EAF as a substitute for coarse aggregate for concrete, 46.67% of the publications reported a decrease in water absorption, the same number (46.67%) reported an increase, and 6.66% reported no impact of these wastes on the water absorption of concrete made from them [35]. In the case of replacing aggregate with sludge, Pizoń et al. [64] observed an increase in the water absorption of concrete with an increase in the content of metallurgical sludge with a grain size of 0–0.25 mm. The water absorption of this reference concrete was 6.5% and that of concrete with the addition of 30, 60, and 90% of metallurgical sludge was 7.4, 7.7, and 8.5%, respectively.

### 3.4. Analysis of the Decrease in Flexural and Compressive Strength after Frost Resistance Tests

A full square RSM model was adopted to analyze the decrease in flexural (DFS) and compressive (DCS) strength after frost resistance tests for two factors (S1 S2 S1 × S1 S2 × S2 S1 × S2). In the case of flexural strength, the analysis of variance showed that statistical significance was met for the two-factor linear model with two-factor interaction (Table S5). After reducing the model using the backward stepwise regression method, a model (S1 S2 S1 × S2) was finally obtained in which all terms were statistically significant. The model’s determination index was R^2^ = 0.92. The coefficients of the fitted model included a constant term (mean process response), linear terms S1 and S2, and the S1 × S2 interaction. The numerical formula (coded values) is Form (6).

DFS = 15.64 − 5.518 · S1 − 0.949 · S2 + 2.266 · S1 · S2(6)

In its current reduced form, the model has only statistically significant terms, while the distribution of residuals is not consistent with the normal distribution (Ryan-Joiner test; *p* < 0.01), and the accuracy is moderate due to the statistical significance of the lack of fit (F = 30.71).

However, in the case of compressive strength, the analysis of variance showed that statistical significance was met for all model terms for the full quadratic model (Table S6). The model’s determination index was R^2^ = 0.96. The coefficients of the fitted model include the complete quadratic model. The numerical formula (coded values) is Form (7).

DSC = 7.24 − 1.156 · S1 + 1.561 · S2 + 0.838 · S1 · S1 − 0.812 · S2 · S2 + 2.000 · S1 · S2(7)

**Figure 6:**
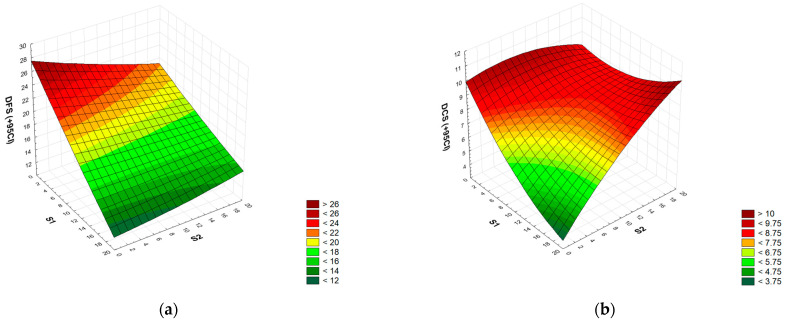
Graph of the dependence of the decrease in flexural strength [MPa] (**a**) and compressive strength [MPa] (**b**) of mortars after frost resistance tests on the amount of added sludge S1 and slag S2 waste {%].

In its current form, the model has only statistically significant terms, the distribution of residuals is consistent with the normal distribution (Ryan-Joiner test; *p* > 0.1), and the accuracy is satisfactory due to the statistical insignificance of the lack of fit (*p* = 0.08). The plots were made for the upper (pessimistic) limit of the 95% confidence interval of strength loss. The dependences on the decrease of the flexural strength (a) and compressive strength (b) of mortars after frost resistance tests on the amount of added S1 and S2 waste are shown in Figure 6. According to the data, all cement mortars modified with the addition of waste sludge and slag show a smaller decrease in flexural strength than the control mortar after frost resistance tests. The flexural strength of the control samples of cement mortars, after 25 cycles of freezing and thawing, is lower by 25.3%, of mortars containing 20% sludge, by 9.2%, and of mortars containing 20% slag, by 18.0%. Mortars containing the addition of a mixture of sludge and slag (20% each) show a decrease in flexural strength by only 11.1%. In the case of compressive strength, it was shown that only cement mortars containing an addition of a mixture of sludge and slag (20% each) show a greater decrease in this parameter (by 9.8%) than the control mortars (a decrease of 8.7%). However, cement mortars containing 20% sludge show a decrease in compressive strength after frost resistance tests by 8.4% and mortars containing 20% slag only by 2.8%. Comparing the obtained results with the literature data is difficult, because tests on the frost resistance of cement mortars with the addition of metallurgical waste have been rarely carried out. Only Santamaria-Vicario et al. [58] reported on the mechanical strength of cement mortars containing EAF slag (grain size 0.063–2.000 mm) after exposure to 56 freeze–thaw cycles. Each cycle of freezing the samples (after 90 days of maturing) was carried out in atmospheric conditions for 6 h at a temperature from −8 °C to −12 °C and then thawed at a temperature from 5 °C to 20 °C. Mortars in which 25 and 50% of the aggregate were replaced with EAF slag showed the same flexural strength as the control mortars (6 MPa). Greater replacement of the aggregate with slag (75 and 100%) resulted in a reduction in the flexural strength of mortars (by approx. 8–10%). However, the compressive strength of the mortars containing 75% of EAF slag was the same as that of the control mortars (25 MPa), while the mortars containing 25% slag showed a higher compressive strength (approx. 5%) and those containing 50 and 100% slag had lower compressive strength (approx. 3–10%) than the control mortars. In the case of concrete, replacing natural aggregate with steelmaking slag has both a positive and negative effect on the mechanical strength of concrete after frost resistance tests. A review [31] showed that 66.7% of the studies reported a positive effect, 25.0% a negative effect, and 8.3% reported no effect of replacing natural aggregate with steelmaking slag on the mechanical strength of cement-based composites. Santamaría et al. [57] showed a negative impact of replacing natural aggregate with EAF slag on the compressive strength of concrete (matured for 90 days) after a frost resistance test (the test included 122 freeze–thaw cycles). After the frost resistance test, concretes in which the natural aggregate was replaced with fine EAF slag (size < 5 mm) showed a decrease in compressive strength (by 33%) and concretes containing EAF with a fine (size < 5 mm) and coarse fraction (size 5–12, 5 mm) a decrease by 25%. However, Arribas et al. [91] showed that replacing natural aggregates with EAF slag, regardless of its grain size (0–5, 5–12, and 12–25 mm), increases the resistance of concrete to the freezing and thawing process. After 158 cycles of freezing and thawing, the compressive strength of concrete with the addition of EAF is 4% higher than that of concrete based on natural aggregate.

### 3.5. Mass Loss Analysis (ML)

A full quadratic RSM model for two factors (S1 S2 S1 × S1 S2 × S2 S1 × S2) was adopted for analysis. The analysis of variance showed that statistical significance was met only for the S1 × S2 interaction (Table S7). After reducing the model using the backward stepwise regression method, a model (S1 × S2) was finally obtained in which all terms were statistically significant. The model’s determination index was R^2^ = 0.25. The coefficients of the fitted model include a constant term (process mean response) and the S1 × S2 interaction. The numerical formula (coded factors) is Form (8).

ML = 0.21 − 0.165 · S1 · S2 (8)

**Figure 7:**
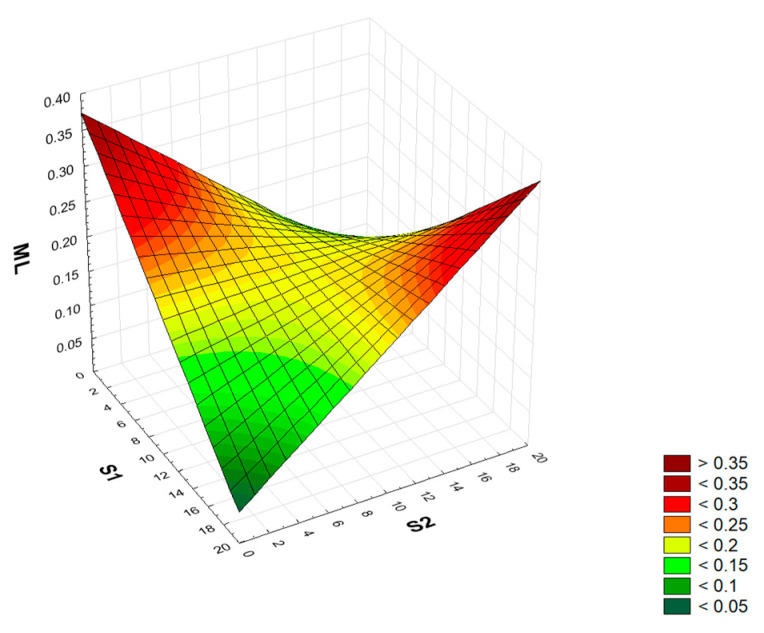
Graph of the dependence of the mass loss [%] of mortars after frost resistance tests on the amount of added S1 and S2 waste [%].

In its current reduced form, the model has only statistically significant terms, the distribution of residuals is marginally inconsistent with the normal distribution (Ryan-Joiner test; *p* = 0.044), and the accuracy is satisfactory due to the lack of statistical significance of the lack of fit. Figure 7 shows the relationship between the mass loss of mortar samples depending on the amount of added sludge (S1) and slag (S2) waste.

All cement mortars modified with the addition of sludge and slag waste show smaller weight loss than the control mortar after frost resistance tests. After 25 cycles of freezing and thawing, the mass loss of control mortars is 0.37%, of mortars containing 20% sludge, 0.1%, and of mortars containing 20% slag, 0.15%. Mortars containing the addition of a mixture of sludge and slag (20% each) show a weight loss of 0.3%. Also, Santarmaria-Vicario et al. [58] reported that mortars in which the natural aggregate was replaced with EAF slag in the range from 25 to 100% (with a grain size of 0.063–2 mm) show a slight weight loss after frost resistance tests (56 freezing/thawing cycles) in relation to the control mortars. Mortars with the addition of 25, 50, 75, and 100% slag showed a weight loss lower by 1.4, 1.2, 1.0, and 1.3%, respectively. Taking into account that the mass loss of mortars was below 3%, it can be concluded that these materials can be classified as frost-resistant materials.

### 3.6. Metal Ion Leaching Test

Research on the properties of mortars or concretes containing waste materials from metallurgical processes mainly concerns their physical and mechanical properties, while the environmental assessment of these products is still incomplete. This important aspect of environmental research is drawn by the work [92], where the correlation was determined between the composition of the slag cements used in composite materials and the degree of immobilization of heavy metals in mineral matrices made of it, and work [90], where the degree of ion leaching was determined by the degree of slag fragmentation (<0.08 mm, <0.5 mm, and <1.6 mm). It should also be emphasized that the process of releasing heavy metal ions from concrete materials may be based on various mechanisms (leaching from the surface, dissolution, percolation, or diffusion) and depends on the environmental conditions during the exploitation (contact with water, soil, sewage, and the presence of aggressive factors) [93].

In this study, a test was performed for leaching metal ions from cement mortars produced with the maximum content of added waste sludge (S1) and slag (S2), assuming that, in this case, the highest content of metal ions in the eluate would be observed. The tests were performed on mortar samples after 28 days of maturing. The test and calculations were performed in accordance with the PN EN 12457 2:2006 standard, assuming no moisture in the samples. Two determinations were made for the tested materials, maintaining the ratio of the volume of the liquid phase (L) to the mass of the solid phase (S) equal to 10:1. After the process of leaching ions from the control samples of cement mortars, the pH of the solution was 10.05, while, in the case of mortars modified with sludge and slag waste, it was 10.28 and 10.20, respectively.

The data presented in Table 4 show that the amount of toxic metal ions, such as Zn^2+^, Cr^6+^, Pb^2+^, and Cu^2+^, washed out from cement mortars containing metallurgical sludge and slag is comparable to the amount of metal ions washed out from the control samples. The obtained concentrations of metal ions in the elution do not exceed the permissible values that must be met when introducing substances that are particularly harmful to the environment into wastewater or land, in accordance with the Regulation of the Minister of Maritime Economy and Inland Navigation of 12 July 2019 (Journal of Laws 2019, item 1311). Therefore, even damaged (cracked) cement composites containing the maximum amount of metallurgical waste in contact with water do not pose a threat to the natural environment. The resulting high level of immobilization of metal ions in the matrix of steelmaking slag-modified cement mortars (BOF) is high. A similarly high level of immobilization of metal ions Cu^2+^, Zn^2+^, and Pb^2+^ was demonstrated by Kuterasińska-Warwas and Król [92] from mortars made on the basis of cement with the addition of silicate fly ash and granulated blast furnace slag. The immobilization level of heavy metals (Cu^2+^, Zn^2+^, and Pb^2+^) ranged from 99.8% to 100%, depending on the age of sample maturation, and of Cr^6+^ ions from 97.73% to 99.71%. With the passage of concrete maturation time, the binding of heavy metal ions in the cement matrix improves, and their lower leachability is observed.

Cement composites made with the addition of silica fly ash and limestone were less effective in immobilizing metal ions than those made with the addition of granulated blast furnace slag. It can be assumed that the effectiveness of heavy metal immobilization in the tested cement mortars depends both on the crystallization of the C-S-H phase, which allows for the incorporation of metal ions into the crystal lattice and on the matrix of the waste itself, because, according to work [94], metals in slag (BF, BOF, and EAF) are closely bound to the slag matrix and are not easily washed out. However, from the research of Roslan et al. [62], it appears that some heavy metals (Pb and Cu) present in the cement slurry can be absorbed by the hydrated C–S–H phases. The metals present in both EAF slag and steelmaking sludge are stabilized into an insoluble form by the interaction between the C–S–H gel and ettringite. The high concentration of leached Fe ions from the cement–sludge slurry (0.022 ppm) indicates that the iron was not completely surrounded by the cement matrix.

### 3.7. Microstructure

**Figure 8:**
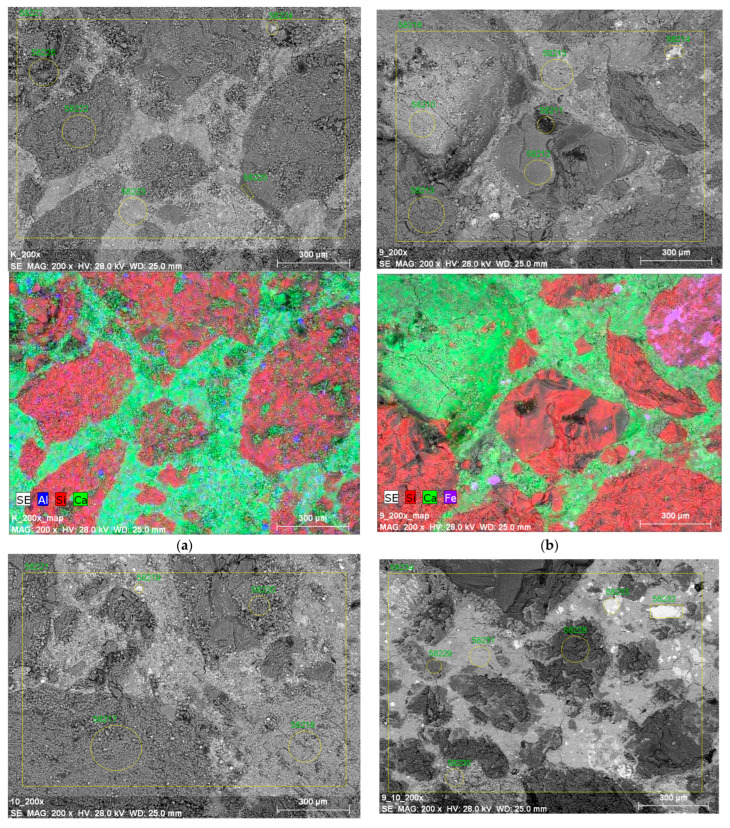

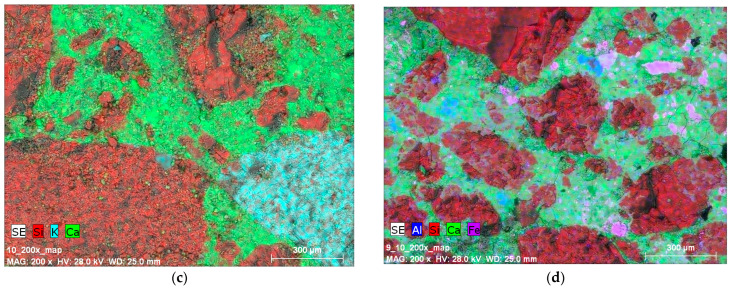
Microstructure of mortars: (**a**) control, (**b**) 20% S1 sludge, (**c**) 20% S2 slag, and (**d**) a mixture of 20% S1 + 20% S2.

The results of observations of the microstructure of samples of cement mortars containing and removing waste after 28 days of maturation are presented in Figure 8. Microscopic images of hardened mortar samples show a homogeneous, compact structure at the boundary between recycled aggregate and cement slurry. One can also observe the even distribution of iron introduced with the addition of waste. Also, Li et al. [95] observed a homogeneous structure of concretes containing steel slag, regardless of the degree of fragmentation of the waste. It was observed that, in concrete, without the addition of steel slag, after 28 days of maturation, there is a large amount of fibrous C–S–H gel, and steel slag micro powder affects the delay in cement setting. Spherical waste particles with a smooth surface did not participate in the hydration process at the early stage of binding.

In turn, Öműr et al. [87] observed a strict structure of mortars containing fresh and weathered (5 years) BOF slag, which contains tobermorite phases with high contents of Ca, Si, Al, and O. Samples of mortars containing natural sand and BOF weathered after the alkaline test (ASR) had a relatively denser microstructure than mortars containing unweathered BOF slag. Meanwhile, the presence of EAF slags has an unfavorable effect on the microstructure of mortars modified with this waste after the crystallization test [58]. Keertan et al. [96] showed that 50% replacement of aggregate with slag provides improved strength and microstructural properties. However, there are no reports on the composition of cement composites with the addition of steel sludge.

## 4. Conclusions

The circular economy and sustainable development require the construction industry to verify its approach to the effective use of waste raw materials as a substitute for natural aggregates. The analysis of the obtained results showed that steelmaking sludge and slag from the production of liquid steel in a basic oxygen furnace (BOF) can be used to produce ecological cement mortars. These mortars can be designed based on designated material models, which allow the design of ecological cement mortars using metallurgical waste (i.e., converter sludge and slag). BOF steelmaking sludge and slag can be safely added in amounts of up to 20% each, and the reduction in compressive and flexural strength does not disqualify this material as a construction product. Mortars modified with waste show very good strength characteristics after frost resistance tests and do not pose any threat to the environment due to the possibility of leaching metal ions from cement matrices. By producing cement mortars, in which sand is replaced by a mixture of sludge and slag (20/20) in the amount of 40% (in total) of the cement mass, we save about 220 kg/m^3^, which, at a price of about 23 EUR/ton, gives us savings of about 5.1 EUR/m^3^ for mortar. Moreover, it should be emphasized that such use of waste allows for saving natural resources and space in landfills and will reduce the costs of depositing waste in landfills.

The widespread use of a mixture of waste sludge and slag from the BOF process (each in the amount of 20% of the cement mass as a sand substitute) for the production of cement mortars, although the obtained results are satisfactory in terms of their mechanical requirements, requires the fulfillment of two elements: firstly, verification of the results for waste materials obtained from other metallurgical plants (determining the extent to which the chemical composition of waste determined by available materials and technology affects the parameters of the produced mortars), and secondly, in the case of some countries (including EU countries), legislative changes in the aspect of allowing waste for the production of construction products. This is necessary due to the classification of sludge to group 10 02 13*, i.e., hazardous waste.

## Figures and Tables

**Table 1:** Chemical composition of the metallurgical waste [%].

Component	Sludge (S1)	Slag (S2)	Component	Sludge (S1)	Slag (S2)
SiO_2_	5.44	9.56	Sr	0.01	0.01
Al_2_O_3_	3.90	5.83	Cr	0.01	0.08
CaO	13.09	38.54	Cu	0.03	-
K_2_O	0.32	0.04	TiO_2_	0.06	0.09
MgO	1.72	5.15	Pb	0.50	-
Fe_2_O_3_	55.66	18.70	V	-	0.03
Na_2_O	0.10	1.07	P	0.08	1.00
Mn	0.46	1.67	SO_3_	1.68	0.43
Ba	-	0.02	Cl^-^	0.70	0.03
Zn	2.47	0.01	Sr	0.01	0.01
SiO_2_	5.44	9.56			

**Table 2:** Actual values of the plan levels and experiment plan without replication.

No.	S1	S2
**Actual values for plan levels**		
1	0	0
2	2.93	2.93
3	10	10
4	17.07	17.07
5	20	20
**Experimental plan without replication**		
1	0	0
2	0	10
3	0	20
4	10	20
5	20	20
6	20	10
7	20	0
8	10	0
9	10	10
10	2.93	2.93
11	2.93	17.07
12	17.07	17.07
13	17.07	2.93

**Table 3:** Measurement results of consistency (C), flexural strength (FS28), compressive strength (CS28) after 28 days, water absorption (WA), mass loss after the frost resistance tests (ML), and decrease in flexural strength (DFS) and compression strength (DCS) after the frost resistance tests of mortars for the assumed experimental plan.

No.	S1	S2	C	FS28	CS28	WA	ML	DFS	DCS
	[%]	[%]	[cm]	[MPa]	[MPa]	[%]	[%]	[%]	[%]
1	0	0	15.90	8.72	50.60	8.72	0.82	25.3	8.7
2	0	10	15.50	7.44	48.09	9.50	0.14	21.4	8.8
3	0	20	14.90	6.89	45.22	10.16	0.15	18.0	8.4
4	10	20	14.60	7.38	43.40	10.20	0.22	16.2	7.6
5	20	20	14.30	7.15	29.41	10.37	0.30	11.1	9.8
6	20	10	14.10	7.51	48.45	9.78	0.17	13.6	6.6
7	20	0	13.65	7.75	51.19	8.78	0.10	9.2	2.8
8	10	0	14.65	8.05	51.55	9.08	0.3	15.8	4.5
9	10	10	15.10	7.42	46.75	9.40	0.11	15.0	7.7
			15.05	7.30	45.07	10.24	0.35	15.4	7.0
			14.85	7.58	46.97	9.17	0.34	14.6	7.2
			15.20	7.36	47.03	9.96	0.16	15.0	7.1
			15.10	7.67	47.25	9.26	0.20	15.3	7.4
			15.15	7.04	46.24	9.71	0.06	15.3	7.6
			15.10	7.02	46.16	9.70	0.09	14.9	7.3
10	2.93	2.93	17.1	7.67	48.10	9.04	0.02	22.0	8.6
11	2.93	17.07	15.55	7.09	43.91	9.77	0.09	18.0	7.8
12	17.07	17.07	15.50	7.26	38.65	10.26	0.17	10.7	8.7
13	17.07	2.93	14.90	8.07	46.18	9.22	0.19	10.4	4.1

**Table 4:** The ion leaching from cement mortar doped with sludge and slag waste.

Element	PK		S1		S2		Limit Values *
	A, mg/dm^3^	s	A, mg/dm^3^	s	A, mg/dm^3^	s	mg/dm^3^
Zn	<0.005	-	0.127	0.049	<0.005	-	2
Pb	<0.005	-	0.022	0.007	<0.005	-	0.5
Cr	0.018	0.009	<0.005	-	0.027	0.009	0.5
Cu	<0.005	-	0.023	0.009	<0.005	-	0.5
Fe	0.251	0.059	0.379	0.058	0.398	0.067	10

A—the released amount of metal ions; s—standard deviation. the * sign can be assumed or added that it (limit) is from the Regulation of the Minister of Maritime Economy and Inland Navigation of 12 July 2019 (Journal of Laws 2019, item 1311).

## Data Availability

The data presented in this study are available on request from the corresponding author (due to privacy).

## Supplementary Materials

The following supporting information can be downloaded at https://www.mdpi.com/article/10.3390/ma17174295/s1: Table S1: Consistency—analysis of variance for the full and reduced models and coefficients of the second-order reduced model; Table S2: FS28—analysis of variance for the full and reduced models and coefficients of the second-order reduced model; Table S3: CS28—analysis of variance for the full and reduced models and coefficients of the second-order reduced model; Table S4: WA—analysis of variance for the full and reduced models and coefficients of the second-order reduced model; Table S5: DFS—analysis of variance for the full and reduced models, and coefficients of the second-order reduced model; Table S6: DCS—analysis of variance for the full and reduced models, and coefficients of the second-order reduced model (coded); Table S7: ML—analysis of variance for the full and reduced models, and coefficients of the second-order reduced model.
