# Supplementary material for: The Application of Converter Sludge and Slag to Produce Ecological Cement Mortars
Source: Materials (Basel). 2024 Aug 30;17(17):4295. doi: 10.3390/ma17174295 (PMC11396167; doi:10.3390/ma17174295)
Supplement: Supplementary file 1 [file materials-17-04295-s001.zip › materials-3172832-supplementary.pdf]

Table S1. Consistency – analysis of variance for the full and reduced models and coefficients of the second-order reduced model.

Source	DF	SS	MS	F	p
Analysis of variance for the full quadratic model					
S1	1	3.88482	3.88482	39.88	0.000
S1·S2	1	0.58151	0.58151	5.97	0.030
S2·S2	1	0.14564	0.14564	1.50	0.243
S1·S1	1	0.09522	0.09522	0.98	0.341
S2	1	0.05409	0.05409	0.56	0.469
Error	13	1.26640	0.09742		
Lack-of-Fit	7	1.19211	0.17030	13.76	0.003
Pure Error	6	0.07429	0.01238		
**Total**	18	6.36289			
Analysis of variance for the reduced model					
S1	1	3.80292	3.80292	35.17	0.000
S1·S2	1	0.61997	0.61997	5.73	0.029
Error	16	1.73014	0.10813		
Lack-of-Fit	10	1.65586	0.16559	13.37	0.002
Pure Error	6	0.07429	0.01238		
**Total**	18	6.36289			
Coefficients of the reduced second-order model (coded)					
**Source**	Coefficient	**SE**	**t**	**p**	
Constant	14.9936	0.0757	198.10	0.000	
S1	-0.715	0.121	-5.93	0.000	
S1xS2	0.362	0.151	2.39	0.029	

Table S2. FS28 – analysis of variance for the full and reduced models and coefficients of the second-order reduced model.

Source	DF	SS	MS	F	p
Analysis of variance for the full quadratic model.					
S2	1	2.08374	2.08374	31.95	0
S1·S2	1	0.24869	0.24869	3.81	0.073
S2·S2	1	0.21437	0.21437	3.29	0.093
S1	1	0.00702	0.00702	0.11	0.748
S1·S1	1	0.00296	0.00296	0.05	0.835
Error	13	0.84792	0.06522		
Lack-of-Fit	7	0.48063	0.06866	1.12	0.452
Pure Error	6	0.36729	0.06121		
**Total**	18	3.48801			
Analysis of variance for the reduced model					
S2	1	2.0837	2.08374	25.23	0.000
Error	17	1.4043	0.08260		
Lack-of-Fit	11	1.0370	0.09427	1.54	0.309
Pure Error	6	0.3673	0.06121		
**Total**	18	3.4880			
Coefficients of the reduced second-order model (coded)					
**Source**	Coefficient	**SE**	**t**	**p**	

Constant	7.4932	0.0659	113.64	0.000
S2	-0.510	0.102	-5.02	0.000

Table S3. CS28 – analysis of variance for the full and reduced models and coefficients of the second-order reduced model.

**Source**	**DF**	**SS**	**MS**	**F**	**p**
Analysis of variance for the full quadratic model.					
S2	1	237.611	237.611	42.38	0
S1·S2	1	65.255	65.255	11.64	0.005
S1	1	49.703	49.703	8.87	0.011
S2·S2	1	6.55	6.55	1.17	0.299
S1·S1	1	1.464	1.464	0.26	0.618
Error	13	72.879	5.606		
Lack-of-Fit	7	69.527	9.932	17.78	0.001
Pure Error	6	3.352	0.559		
**Total**	18	438.976			
Analysis of variance for the reduced model					
S2	1	237.611	237.611	41.25	0.000
S1·S2	1	65.255	65.255	11.33	0.004
S1	1	49.703	49.703	8.63	0.010
Error	15	86.407	5.760		
Lack-of-Fit	9	83.055	9.228	16.52	0.001
Pure Error	6	3.352	0.559		
**Total**	18	438.976			
Coefficients of the reduced second-order model (coded)					
**Source**	Coefficient	**SE**	**t**	**p**	
Constant	45.798	0.551	83.18	0.000	
S1	-2.493	0.849	-2.94	0.010	
S2	-5.450	0.849	-6.42	0.000	
S1xS2	-3.61	1.07	-3.37	0.004	

Table S4. WA – analysis of variance for the full and reduced models and coefficients of the second-order reduced model.

**Source**	**DF**	**SS**	**MS**	**F**	**p**
Analysis of variance for the full quadratic model					
S2	1	3.64715	3.64715	46.64	0.000
S1	1	0.131	0.131	1.68	0.218
S1·S2	1	0.0186	0.0186	0.24	0.634
S1·S1	1	0.01014	0.01014	0.13	0.725
S2·S2	1	0.01014	0.01014	0.13	0.725
Error	13	1.01648	0.07819		
Lack-of-Fit	7	0.12291	0.01756	0.12	0.994
Pure Error	6	0.89357	0.14893		
**Total**	18	4.85006			
Analysis of variance for the reduced model					
S2	1	3.6472	3.64715	51.54	0.000
Error	17	1.2029	0.07076		
Lack-of-Fit	11	0.3093	0.02812	0.19	0.991

Pure Error	6	0.8936	0.14893		
**Total**	18	4.8501			
Coefficients of the reduced second-order model (coded)					
**Source**	Coefficient	**SE**	**t**	**p**	
Constant	9.60	0.0610	157.24	0.000	
S2	0.6752	0.0941	7.18	0.000	

Table S5. DFS – analysis of variance for the full and reduced models and coefficients of the second-order reduced model.

**Source**	**DF**	**SS**	**MS**	**F**	**p**
Analysis of variance for the full quadratic model.					
S1	1	243.605	243.605	178.96	0.000
S1·S2	1	25.674	25.674	18.86	0.001
S2	1	7.197	7.197	5.29	0.039
S1·S1	1	4.345	4.345	3.19	0.097
S2·S2	1	0.212	0.212	0.16	0.699
Error	13	17.696	1.361		
Lack-of-Fit	7	17.222	2.460	31.12	0.000
Pure Error	6	0.474	0.079		
**Total**	18	298.800			
Analysis of variance for the reduced model					
S1	1	243.605	243.605	163.68	0.000
S1·S2	1	25.674	25.674	17.25	0.001
S2	1	7.197	7.197	4.84	0.044
Error	15	22.324	1.488		
Lack-of-Fit	9	21.850	2.428	30.71	0.000
Pure Error	6	0.474	0.079		
**Total**	18	298.800			
Coefficients of the reduced second-order model (coded)					
**Source**	Coefficient	**SE**	**t**	**p**	
Constant	15.639	0.280	55.88	0.000	
S1	-5.518	0.431	-12.79	0.000	
S2	-0.949	0.431	-2.20	0.044	
S1xS2	2.266	0.546	4.15	0.001	

Table S6. DCS – analysis of variance for the full and reduced models and coefficients of the second-order reduced model (coded).

**Source**	**DF**	**SS**	**MS**	**F**	**p**
Analysis of variance for the full quadratic model.					
S1·S2	1	19.9992	19.9992	134.86	0
S2	1	19.4909	19.4909	131.43	0
S1	1	10.685	10.685	72.05	0
S1·S1	1	2.0379	2.0379	13.74	0.003
S2·S2	1	1.9088	1.9088	12.87	0.003
Error	13	1.9279	0.1483		
Lack-of-Fit	7	1.5336	0.2191	3.33	0.082
Pure Error	6	0.3943	0.0657		

**Total**	18	54.8274			
Coefficients of the reduced second-order model (coded)					
**Source**	Coefficient	**SE**	**t**	**p**	
Constant	7.236	0.133	54.24	0.000	
S1	-1.156	0.136	-8.49	0.000	
S2	1.561	0.136	11.46	0.000	
S1·S1	0.838	0.226	3.71	0.003	
S2·S2	-0.812	0.226	-3.59	0.003	
S1·S2	2.000	0.172	11.61	0.000	

Table S7. ML – analysis of variance for the full and reduced models and coefficients of the second-order reduced model.

**Source**	**DF**	**SS**	**MS**	**F**	**p**
Analysis of variance for the full quadratic model					
S1·S2	1	0.136146	0.136146	5.76	0.032
S2·S2	1	0.040299	0.040299	1.7	0.214
S2	1	0.033111	0.033111	1.4	0.258
S1	1	0.016495	0.016495	0.7	0.419
S1·S1	1	0.000483	0.000483	0.02	0.888
Error	13	0.307279	0.023637		
Lack-of-Fit	7	0.224936	0.032134	2.34	0.160
Pure Error	6	0.082343	0.013724		
**Total**	18	0.549095			
Analysis of variance for the reduced model					
S1·S2	1	0.13615	0.13615	5.60	0.030
Error	17	0.41295	0.02429		
Lack-of-Fit	11	0.33061	0.03006	2.19	0.174
Pure Error	6	0.08234	0.01372		
**Total**	18	0.54909			
Coefficients of the reduced second-order model (coded)					
**Source**	Coefficient	**SE**	**t**	**p**	
Constant	0.2095	0.0358	5.86	0.000	
S1xS2	0.1650	0.0697	2.37	0.030	
